# “That Is What We Have Left of Her”: The Significance of Transitional Objects After the Death of an Infant in a Norwegian Context

**DOI:** 10.1177/10497323241271920

**Published:** 2024-09-14

**Authors:** Inger Emilie Værland, Anne Beth Gilja Johansen, Marta Høyland Lavik

**Affiliations:** 1 Pediatric Department, 60496 Stavanger University Hospital, Stavanger, Norway; 2 The Research Group for Nursing- and Healthcare Science, 60496 Stavanger University Hospital, Stavanger, Norway; 3 Professional Relations in Health and Welfare, University of Stavanger, Stavanger, Norway; 4 Chaplaincy Department, 60496 Stavanger University Hospital, Stavanger, Norway; 5 Faculty of Theology, Stellenbosch University, Stellenbosch, South Africa

**Keywords:** tangible memories, transitional objects, memory making, continuing bonds, relational autonomy, grief, parents, infants

## Abstract

When an infant dies in a neonatal intensive care unit in Norway, healthcare professionals provide bereaved parents with objects intended to help them processing their loss. Such objects can be clothes, blankets, soft animal toys, hand- and footprints, hair, as well as scrapbooks where the short life is documented through text and photo. By interviewing bereaved parents in three focus groups, we investigated the parents’ use of these objects. Applying the method of reflexive thematic analysis, we developed three themes from the data material: (i) the importance of preserving objects, (ii) the approach to the objects, and (iii) the ambivalence concerning the objects. Pertinent to all themes was the parents’ feeling of ambivalence toward the objects. On the one hand, the parents experienced the objects to affirm parenthood and manifest that the infant existed as a family member. Further, the objects were important in ritualization while according the child its status as deceased. Also, the objects helped the bereaved establish and keep continuing bonds with the deceased and to integrate their traumatic experience of losing a child. On the other hand, the bereaved parents shared that they were ambivalent toward the objects as they stirred up both good and painful emotions. The objects reminded them of their shocking and traumatic loss and the bereaved did not want to be confronted with this all the time. Therefore, through a preference for some objects and indifference toward others as time passed, the parents worked on transforming their bonds with the lost infant.

## Introduction

Some parents experience the death of their infants during pregnancy, shortly after birth, or when children are young. A study by Butler et al. (2015) showed that to incorporate the passing of a child in their lives, objects that belonged to or were related to the infant are important to the bereaved. In general, tangible memories in this sense are often objects that have belonged to or can symbolize the deceased (Mathijssen, 2018). Such objects may also be named melancholy objects as they represent the memorialized objects of mourning (Gibson, 2004). Tangible memories can also be called transitional objects (LeDuff et al., 2017). This is the term we have chosen to use in this article. The terminology, “transitional objects,” is associated with the observation that children in their first year of development use inanimate objects to facilitate the transition away from the mother (Winnicott, 1991). In our empirical study from a Norwegian context, the parents mention items such as blankets, clothes, hand- and footprints, hair, memory books and boxes, necklaces with names, keys, photos, soft animal toys, and gravestones as important objects when they try to come to terms with their loss (Værland et al., 2021).

In Norway, parents who lose an infant are encouraged to see, touch, and hold the child. In this way, healthcare professionals in hospitals encourage bereaved parents to face and process the loss and assist them in making memories of the dead infant. In the Norwegian setting, this proactive approach toward mourning over deaths at the very onset of life has among healthcare professionals only existed during the past few decades. In 1997, a new law was passed which guaranteed every citizen, regardless of age, the right to be buried in an individual grave (Lov om gravplasser, kremasjon og gravferd [gravplassloven] [Act on Cemeteries, Cremation and Burials (Cemeteries Act)], 1996). Before 1997, infant deaths were not widely recognized as legitimate causes for mourning, and infants who died in hospitals were placed either together with a random body of an adult and buried anonymously in this individual’s casket (without the knowledge of the adult’s next of kin and the bereaved parents) or handled as organic material and disposed of accordingly (Kristvik, 2018, pp. 106–109). As late as 2006, parents got the right to know where their dead infant was buried (Opplysninger om dødfødte barns grav [Information about stillborn infants’ graves], 2006). Today, the death of an infant is regarded as a legitimate death, and mourning the loss is not only accepted but also encouraged by healthcare professionals and society at large. However, despite these developments, grief following infant loss is complex and can in contemporary Western cultures be experienced as a condition to overcome and a situation often accompanied by a sociologically ambivalent moral framework (Christensen et al., 2019, pp. 81–83; Kofod & Brinkmann, 2017). The status of a stillborn infant has changed in Western cultures during recent decades. In recent times in the Czech Republic, an infant was regarded as biological waste and denied a funeral as a human being (Smidova, 2019). In areas where the Roman Catholic Church is dominant, stillborn babies were buried anonymously in unconsecrated grounds. After 2000, monuments were made to stillborn infants that did not have a grave or a memorial place of their own (Faro, 2021).

To commemorate the infants’ biological body, life, and death, parents create ritualizations that incorporate physical objects, which situates the infant in a wider context (Christensen et al., 2019). Memory making when an infant dies can be regarded as ritualization that serves several functions (Værland et al., 2021). The overall function of memory making is to create evidence of the infant’s life, but also to affirm parenthood (Thornton et al., 2020). According to Vig et al. (2021), parents cope better when remembering the deceased infant on birthdays or death anniversaries. Objects such as the child’s possessions, photos, footprints, locks of hair, and “memory boxes” are important to validate the child as a member of the family. Currie et al. (2019) find that transitional objects can stimulate loss-focused behavior and help parents cope with loss. Some parents try to minimize looking at these objects, while others treasure the memories they evoke. A recent study found that memories evoked by objects are an important support in the bereavement process, and parents expressed their love for their infant when describing or presenting these objects (Lakhani et al., 2023). Parents who experienced stillbirth continued bonds with the infant through objects (Burgess et al., 2023). While displaying objects can be important for some parents, others find it difficult to face items that remind them of their diseased infant (Murphy & Thomas, 2013). Memory making is important, even when the infant is not viable by being born just before 24 weeks of gestation (Smith et al., 2020). In their meta-synthesis, Kingdon et al. (2015) find that there are different ways healthcare professionals can guide parents to make good decisions in facing their unknown and grief-stricken situation. However, studies have established that parents who lose their infants find it awkward when health professionals photographed them and their infants. However, retrospectively, most were pleased it was done (Abraham & Hendriks, 2017). According to Fuller and Kuberska (2022), various objects can have special meaning after pregnancy loss. These objects are not necessarily a part of bereavement care as memory boxes. In a previous work, we interviewed healthcare professionals and members of a parents’ support group about the act of making memories when an infant dies. We saw that the act of memory making was a kind of ritualization for the providers and that the objects were intended to be used to help parents in the grieving process (Værland et al., 2021). The aim of the present study is to explore the use of transitional objects when remembering a dead infant and suggest the significance these objects may have for the bereaved.

### Theoretical Framework

Qualitative research is always context bound, positioned, and situated. Our underlying research values and assumptions are strongly linked to our professional backgrounds as nurse and theologians. Common for all three of us in the process of engaging with the data was a hermeneutical basis for knowledge production. Through our interdisciplinary discussions, we experienced our personal and professional prejudices and preunderstandings as resources that enable a thick interpretation of the data material and ourselves. In the process of analysis, we have negotiated the development of themes and how to interpret these. By making our researcher subjectivities visible here, the reader can evaluate how these shape our analysis (Braun & Clarke, 2023, p. 700). The specific theories that inform our interpretation of the data material are bereavement, grief, trauma, and ritual.

### Bereavement and Grief: Continuing Bonds

There has been some controversy in the bereavement literature as to whether the process and/or purpose of grief work involves letting go or maintaining bonds with the deceased person (Stroebe & Schut, 2005). Early theorists, such as Bowlby, Parkes, and Kübler-Ross, often treat grief prescriptively, as a sequence of stages through which each individual must pass to recover from a “maladaptive” situation (Stroebe, 2002). This does not mean that the deceased should be forgotten or dismissed altogether, but that they do occupy a place in the past. They should have a place in memory but should not play an active role in the future lives of the bereaved (Mathijssen, 2018; Rosenblatt, 1996; Stroebe & Schut, 2005). However, since the 1990s, the academic discourse has focused on continuing bonds (Klass et al., 1996, pp. 844–845): “We were responding in *Continuing Bonds* (Klass et al., 1996) to the fact that for much of the 20th century continuing bonds had been regarded an indicator of pathology in grief. We wanted to show that interacting with the dead could be normal rather than pathological” (Klass, 2006, pp. 2–3). Continuing bonds can be understood as a relational and dynamic process of absence and presence. Memories can symbolize and arouse the deceased as an absent presence. They can act as a channel for continuing the relationship and interpreting and processing the trauma. Transitional objects can be placed outside typical places for memorialization (Maddrell, 2013). Bonds to the dead person can be tightened and loosened by moving different objects in and out of particular spaces. The belongings of the deceased are gradually separated, transformed, and incorporated, both in distant spaces and in the daily environment of the bereaved. These practices can be considered bereavement rituals. By moving and making a hierarchy of the value of the objects, the bereaved form and reinforce transformation in their bonds with the dead (Mathijssen, 2018).

### Bereavement and Trauma

The original meaning of the Greek word trauma refers to an open physical wound or damage to the skin (Blindheim, 2014). The term “psychic trauma” comes from Eulenburg (Nijenhuis, 2015, p. 9) and refers to the meaning of wound, injury, or shock in a psychological sense. The term therefore refers in both of these contexts to the effect an event has on a person and not to the event itself (Blindheim, 2014). Bereavement can cause trauma: “Bereavements that occur under external traumatic circumstances increase the risk for dysfunction, trauma symptomatology, as well as disordered and prolonged grief” (Rubin et al., 2020, p. 1). According to Christiansen et al. (2013, p. 605), “[t]he death of a child has been reported to result in significantly higher intensities of grief than the death of a spouse or a parent and often leads to complicated grief.” Blindheim (2014) explains that lack of integration of the experience is central when it comes to the effect of the traumatic event. The force of what has happened can be so powerful that one is unable to integrate the event into one’s autobiographical history. He refers to Nijenhuis, who defines trauma as follows: “The essence of this injury is a lack of integration of the phenomenal experience and phenomenal event in the survivor’s personality” (Nijenhuis, 2015, p. 270).

### Rituals

Rituals that are related to birth, maturity, reproduction, and death are ceremonies that mark an individual’s transition from one status to another. Such rituals are common in all cultures, and Arnold van Gennep was the first to coin these as rites of passage (*rites de passage*). According to van Gennep, the rites can be subdivided into rites of separation (preliminal rites), transition rites (liminal rites), and rites of incorporation (postliminal rites) (van Gennep, 1960, pp. 10–11). Victor W. Turner further developed the concept of liminality and argued that liminality is a condition in the transition phase where the individual is “betwixt and between”—no longer classified in the former status and still not yet classified in the new status (Turner, 1996, p. 511). This indicates that the liminal phase can be regarded as an ontological and socially insecure and unstable position to be in (Grimes, 2000, p. 6; Jørgensen et al., 2021, p. 102). That also makes it a critical phase as it raises the question whether one will be acknowledged and incorporated into the new state or not (Værland et al., 2021, p. 2) When an infant dies, there is often a close proximity between the phases of birth and death, and the rituals and ceremonies that usually accompany birth ought to be replaced by those accompanying death.

The following broad definition shows how the performative and individual sides of rituals gradually have become more emphasized:

Very generally, ritual is any activity—sacred or secular, public or private, formal or informal, traditional or newly created, scripted or improvised, communal or solitary, prescribed or self-designed, repeated or one-time only, that includes the symbolic expression of a combination of emotions, thoughts, and/or spiritual beliefs of the participant(s) and that has special meaning for the participant(s). (Castle & Phillips, 2003, p. 43)

As described in our previous article (Værland et al., 2021), it is common to distinguish between ritual and ritualization. A ritual is often conceived as a text or formula used in a religious context or in other contexts where something needs to be expressed symbolically. By ritualizing, we mean symbolic activities people take part in to derive meaning. In ritualizing, people can use written rituals but can also create new symbolic activities they use in their meaning-making efforts (Danbolt & Stifoss-Hanssen, 2017, p. 355). According to the religious studies scholar Catherine Bell, ritualization must be understood in connection with context, as it “[. . . ] always takes place within a larger and very immediate sociocultural situation” (Bell & Jonte-Pace, 2009, p. 100).

We are not investigating rituals and ritualization within traditional religious contexts. Our interest lies in understanding how the transitional objects can function both as rites of passage and as ritualized acts. The terminology “ritualized acts” is taken from Bell. By introducing this term, she goes beyond former definitions of ritual in defining ritual as “the production of ritualized acts” (Bell & Jonte-Pace, 2009, p. 140).

## Method

This empirical study has a qualitative, explorative design. Focus group interviews were performed and a semi-structured interview guide was compiled to examine the parents’ experience and use of objects (Krueger & Casey, 2015, pp. 44–46). The interviews were recorded and transcribed verbatim.

### Sample

In recruiting participants, we contacted a parent support group. In Norway, there is a nationwide association for bereaved families who have experienced perinatal loss or the loss of toddlers. We also contacted a leader of a bereavement group for parents in a hospital in Norway. Altogether 20 bereaved parents, 16 mothers and four fathers, were included in the study. They experienced their loss between 1 month and 22 years before the interviews took place. They had experienced the loss of an infant from the gestational age of 20 weeks to 18 months. At the time of data collection, the parents’ age was from 23 to 59 years, with a median age of 34. Two parents had lost non-viable infants below the gestational age of 23 weeks. Five parents had experienced stillbirth between the gestational ages of 27 and 41 weeks. Six had lost their infant within the first day after birth, and the gestation age of these infants was between weeks 24 and 41. Six of the parents experienced the loss of their infants between the gestational ages of 23 and 37 weeks. These infants had lived from 2 to 10 days. Two participants lost toddlers that were approximately 18 months old. Two participants had experienced two losses. One infant had died at home and the others in hospitals.

### Data Collection

The data were collected through three focus group interviews in southern Norway. Focus groups are a way of exploring people’s views and how social interactions shape these views (Tritter & Landstad, 2020). The interview guide was based on the recommendations of Krueger and Casey (2015). The interview started with a presentation of the researchers and parents. The introductory question dealt with the significance of objects of memories and how important they were in the bereavement process. The key questions focused on what the transitional objects were like, the bereaved’s relation to them, the aspect of time, as well as acknowledging the infant and parenthood. During discussions, the parents gave us new knowledge about the significance of the objects. Two of the researchers conducted all the interviews; one moderated the conversation, while the other asked additional questions and summarized the answers. The third researcher became involved in the process of writing the article. The researchers present at the focus groups interviews recorded and transcribed the interviews verbatim. Each group was interviewed once, and each interview lasted approximately 1 hr.

### Reflexive Thematic Analysis

Reflexive thematic analysis (Braun & Clarke, 2006, 2019) was used to construct, describe, and analyze themes developed from the data. The process of developing codes and themes was collaborative and reflexive, as we all came to the research process with our own professional preunderstandings and assumptions (Braun & Clarke, 2019, 2023). The themes were thus “generated at the intersection of the data and the researcher’s positioning, skill and (considerable) interpretative labour” (Braun & Clarke, 2023, p. 700). The process was organic as we went back and forth between the following six phases: (1) First, the researchers studied the dataset thoroughly. (2) Temporary codes were created across the dataset. Our approach was deductive as well as inductive. We were influenced by the theoretical approach from a former study (Værland et al., 2021), as well as our professional background as chaplains and nurse. We were also aware that the findings could be illuminated by theory after the analysis (Dahlberg & Dahlberg, 2019). (3) The codes were organized into temporary themes. (4) The temporary themes were further examined and reorganized. (5) We tried to identify the core of every theme, naming them, as well as considering each theme in relation to the others. (6) Finally, the article was written.

### Ethical Considerations

When we ask about the significance of objects after the death of an infant, we are aware our research goes into the painful past of the participants, and that the parents will recall feelings of an existential character. We emphasized that the participants should not expose themselves by sharing more than they were comfortable with. Some of the participants were familiar with the other participants’ stories. The study followed the Helsinki Declaration (World Medical Association, 2022). The participants gave their written consent to participate in the study and were informed they could withdraw their participation at any time. Codes with the name, age, etc. of the participants were stored in a secure research server at the hospital trust, and all the names used in this article are pseudonyms. The Norwegian Centre for Research Data (NSD) approved the study (Ref. No: 791685).

## Results

The objects had significance for the parents, and our thematic constructs of what the parents shared in this respect are presented in the three following themes: (i) the importance of preserving objects, (ii) the approach to the objects, and (iii) the ambivalence concerning the objects.

### The Importance of Preserving Objects

Many of the parents were guided by healthcare professionals in making memories, and they were grateful for all the objects they brought from the hospital. Those who received little guidance wished that someone had taken more responsibility for preserving objects of their lost children.

### They tell you what you should do

The parents said they were in a state of shock and disbelief when their infants died. The staff supported and guided them so that memories were made and preserved. The staff took photos, printed them, and wrote in memory books. The midwife supporting Alexandra borrowed her mobile phone and took many photos. Alexandra said:

I wondered why she (the midwife) was taking photos … anyway, I am happy about it now.

The parents emphasized the importance of someone taking responsibility for creating memories, encouraging, or even persuading the parents to do so. Beth explained it this way:

We have quite a few different objects. The most important to us are the photos, which were taken by nurses and midwives who were there when he died, when we did not want to see him. Those are really the most important ones that we have. We were persuaded to take those photos, and to take even more photos.

Several other parents had similar experiences about the role of the healthcare professionals. For instance, Lise said:

You just want to bury your head in the sand and forget about it. It is important that they are there, take photos, make foot- and handprints; tell you what you should do, because you will think this is good later. It was a lot like “you have to do that,” “no, I don’t want to,” “yes, you should” … There will come a time afterwards when you will need it.

Some of the parents who had children born alive also got memory books/diaries, in addition to other objects. These books made by the staff were of great significance, as Celine explained:

We got a diary, and that diary is important, with photos that they have taken. They wrote a little every day or at every shift. Both about her, about us, and about everyone who stopped by. It is important (…) the hand- and footprint that they made; I remember that when we were going to pick it up (…) I got my hands on it first, but I could not carry it as I was so afraid of losing it. My husband had to take it. Right then I could not handle it, because I was so afraid of destroying it.

Monica also appreciated the book made by the staff:

At first, we spent a couple of hours at a NICU. During those hours, the staff managed to make a memory book, and it has helped us a lot. I read it almost every week, often every day.

### I wish somebody had taken the responsibility

One mother, Alice, said the staff did not take photos, and she was concerned that healthcare professionals should remember to encourage parents to take photos:

They didn’t take photos and they didn’t do anything. Not until the last nurse, when we sat and held him after everything was turned off; then she asked, could we take … shall I take … photos of you? Moreover, I can feel that ... that no one said it was okay to take pictures of him.

Another mother had a similar experience after a stillbirth, with only a few photos from the hospital. She said:

I wish the midwives would just have taken some responsibility and taken more photos. I do not know if they have a camera or what, but if they do not, they should have picked up our phones and just have done it. Because that is not what you are thinking about then.

The parents shared that after coming home, they had made different kinds of tangible objects. They made memory books and memory boxes containing different objects belonging to their infant. Emily lost her 18-month-old child several years ago. She did not get any memory box at the hospital as parents may get nowadays, but she collected some objects herself:

There was nothing like that from the hospital, but I have collected things myself. For example, we have a box hanging on the wall with his shoes, a pacifier, the toothbrush, the pyjamas. (…) We also have a separate box at home with lots of photos.

All the parents emphasized in different ways that the objects were very important to them. Beth proclaimed that without the photos, she would have been depressed for the rest of her life. With a small laugh, she added:

I save them before I save my husband.

Alice explained that without the memory objects, she would have experienced double sorrow:

Then you have nothing to turn to when you are having a hard time.

### The Approach to the Objects

Parenthood is affirmed through the objects, and they manifest that the infant existed as a member of the family.

#### … As If She Is Here …

As we have seen, some parents create their own objects of memory. They said these objects brought their child closer to the family. Monica said:

I made a photo book out of all this, and I wrote a story in which I treat her as if she is here.

Monica described how she wrote to the child at the hospital about their first trip with a pram and who came to visit them. She takes this book with her to the grave and reads aloud from it to the buried child. When family members visit, she brings the book with her, and they read it together.

Some parents made family portraits that included the lost infant.

Beth said:

I made a family portrait myself. (…) There he is like a shadow. He is there, with silhouette and everything. Because it is a black and white photo, he is white, and the rest of us are just silhouettes in black. (…) it is a memory that means a lot.

Another parent, Alice, made a photo with the deceased infant’s siblings:

So, we have made it like he is a shadow. That photo means everything; because it is the only photo we have of all three.

Several of the parents mentioned the importance of teddy bears that belonged to their child. Celine said their daughter had two teddy bears, a small one and a big one. The small one accompanied her to the grave, while the big one remained with her parents:

It was brand new. Eventually it is well used. In the beginning, it was included, no matter what we did. On holidays, it has been in Turkey, it has been all over the place. Now it is not with us all the time, but it is still with us ... Belinda’s teddy bear has almost its own life.

#### … She Was Wearing It, She Had It Around Her…

Objects that were physically connected to the infant seem to be of special importance. Monica explained:

To me, it is special that she was there, she was wearing it (a blanket), and she had it around her. We were in the room when that photo was taken. She was alive when that photo was taken. She has trodden in that footprint. That is what we have left of her. Except for, in a way our love, and that she remains in our hearts and in our minds, and the tombstone.

The items are an important part of the parents’ lives. Asked what the objects related to their deceased infants mean, Alice answered:

Everything. I have vacuum-packed his duvet and his cuddly blanket. I am terrified that his scent will disappear when I one day feel like opening it.

Beth reflected similarly to that question:

I think it is a bit difficult to answer exactly what it means. Because it is as you say, that [it] is kind of everything; that is what we have. (…). It was his life, those things; he should have worn them, and the presents he got, and yes, it is a big part of him and the one he was supposed to be, if I may say so.

#### … He Is Always in My Heart

How the parents relate to the objects seems to change as time passes. In the beginning, it is important to be close to the items as often as possible. John explained:

99% of the time we spent looking at the photos, thinking and talking about him. Anyway, you change … and perhaps choose some favourite items and find other ways of doing it … we spend more time at the grave than looking in the memory box and smelling things, compared to what we did at first.

This is also apparent in relation to the infant’s grave. Several parents described how initially they felt that they wanted or needed to visit the grave almost every day. Emily says:

At first, I had to go up to the cemetery every day and do something physical because I had to be near him. Now it’s not like that anymore. Now I think that he is always in my heart, and I think of him very often.

Even though they visit the grave more rarely now, the grave is still very important. Alice said:

I have been thinking about it. I could not have moved because of the grave. (Assent from the others). Not now, not so soon after. Maybe never.

Alice also reflected on the fact that the way her family relates to the grave has changed over time. Initially, they went there almost every day and felt guilty if they went away for a few days. As time passed, the grave has become a place to remember her lost infant. She said:

Now it is a very nice memory. The grave, if I may say so. There is a little Winnie the Pooh carved into the stone, so Winnie the Pooh has somehow become our thing. Then, each time I think of Winnie the Pooh, I think of him. In a way, when there are memories like that, for me at least, it is more part of everyday life. It is a constant reminder; not that I need to be reminded of it, but it is such a nice reminder. So that yes, if I see Winnie the Pooh on some t-shirt in some shop, it immediately pops into my head... so I think it’s nice.

Other participants also reported a change in the frequency of looking at the photos, from all the time to less frequently as time passed. Alice said:

I have them on my phone and look at them from time to time … and especially in the beginning, when I was able to sort of look at memories and was able to look at the photos, I was looking at them all the time.

### The Ambivalence Concerning the Objects

Looking at photos and other objects can be comforting or upsetting, depending on whether it is a good or bad day.

#### It Is a Consolation, and It Hurts

The participants expressed diverse views toward the objects of memory. While the items were important, they could also arouse difficult feelings. Some objects were easy to relate to, while others were more challenging. Time and mood influenced the experience of the object. Astrid explained her experience:

It is a consolation, and it hurts. In a way, you see who you made … it depends on where you are; what kind of day you have, whether it is good or bad.

The participants acknowledged the importance of the objects of memory, even though it was sometimes challenging to face them. The participants described addressing grief in different ways. On tough days, some pursued the feeling of grief. When it was hard to endure, it helped the participants go deeper into it and confront the loss. Alice said:

I have some films that I hesitate to look at. I have seen them, but on hard days only, when I want it even harder. To come through it, I can watch those films.

Different objects can evoke different feelings. Some items provide comfort, while others arouse challenging feelings. For example, it can be difficult to relate to clothes that were never worn, while blankets and memory books are looked at frequently. Celine described it this way:

It’s too demanding to display the things, like Belinda’s hat. We know where it is when things are hard. There is still a little hint of her in it, her scent. The other days it can be where it belongs, out of sight. Anyway, the teddy bear sits on the sofa.

Most of the participants said they had objects displayed in their homes. Items that had belonged or should belong to the infant were sometimes removed and later put back again.

#### They Know Her Through Our Histories, Our Photos, and Our Memories

The objects are important as a means to remember and maintain the infant as a member of the family. One way to maintain these memories, and to let others be acquainted with the infants, is to display photos and other objects. Some parents experienced their loss during the pandemic, and a few had seen the infant. Sharing their memories with others reminds the parents that the infant has been around. Monica said:

Family and friends express that they wish that they knew her. However, they feel that they know her through our stories, our photos and our memories. When they have seen something tangible—as the christening dress; it is so small; she was so small, it was almost too big—you get an understanding of who she was.

Some objects were placed where guests could see them. Linda said:

I always hope that visitors will watch; however, few take the initiative to do so.

Some participants were hesitant to speak about the deceased infant, depending on how they felt on a particular day:

You never get the reaction you want, anyway.

The parents said they felt a great responsibility to keep alive their memory of the infant. Alice was concerned her infant’s belongings should be stored in a dignified way. She took some time to find boxes that were appropriate because she was concerned about what might happen to the belongings in the future:

What I am most afraid of is that my husband and I should die at the same time. Moreover, when they clear out after us, they will throw our things in a container. The worst is that they will throw away his things as well.

Some participants were not sure whether the objects helped them feel like parents. Anyway, in the interviews, they emphasized they *were*, not *had been*, parents. Beth explained:

The tangible memories made by our family do that. I feel like I am the mother of this infant. Even the porcelain figure, which I initially did not like.

The siblings include the deceased infant in the family. The deceased infant’s siblings talk about their deceased brother or sister and visit the grave. Some siblings are eager to look at photos and the memory book. Helen told us her daughter loved looking at a small book with photos:

It is with her other toys, and she knows who all the people are. It is annoying for visitors who do not necessarily want to watch. Anyway, I appreciate it. The only memory we made ourselves is used daily.

## Discussion

From the results above, it is evident the parents are grateful for the objects healthcare professionals have made in memory of their children. At the same time, it is clear they have ambivalent feelings toward these items.

### The Function of the Objects

In our interpretation, the objects are transitional objects, and as such they seem to have a fourfold function for the bereaved. First, they guide and help the parents through the liminal phases. From the theory on rites of passage (van Gennep, 1960, pp. 10–11), we know that all cultures have developed rituals to help individuals and communities with major transitions in life (birth, initiation, reproduction, and death). According to Turner, the liminal phase—when one is no longer classified in the former status, but yet not classified in the new one—is an ontologically and socially unstable position to be in (Grimes, 2000, p. 6; Jørgensen et al., 2021, p. 3; Turner, 1996, p. 511). According to Winnicott, transitional objects generally refer to inanimate objects that relate to an infant’s transition away from the mother (Winnicott, 1991). Winnicott’s insights can also be applied when it comes to the transition from life to death and when we interpret what function objects have for the child’s bereaved parents. When parents experience that their child goes through the ultimate transition from life to death, they experience the phases of separation, transition, and incorporation on behalf of themselves and the child. In this process, the transitional objects help them incorporate the child in its new status. These objects also have the potential to give them psychological strength, consolation, and a feeling of security and stability. Transitional objects in this sense are mementos that also confirm the meaning of parenthood, even when the infant had a short life (LeDuff et al., 2017).

Second, and connected with the first function, is our contention that transitional objects are important elements in the bereaved’s ritualization around giving the child status as a family member and at the same time acknowledging its status as deceased. In our data material, birth and death has happened in relatively close proximity (the oldest child died at the age of 18 months). Besides, societal rites around birth and death performed by the participants in the present study are more private rituals, such as making and actively using objects of the dead child in the process of grief. These privately invented rituals are born out of a specific situation of loss and are examples of how human beings create rituals to help them in the process of giving the child its new status. The abrupt ending of a new life makes the transition from one status to another difficult to overcome. Apart from participating in traditional ceremonies that usually mark these transitions, parents may sometimes feel they and the infant are left betwixt and between. To leave the unstable phase of liminality and transit into the status of parenthood of a deceased infant, the parents work on creating continuing bonds with the deceased child, and the objects assist them in this. Through memory making, they incorporate the infant among the dead at the same time as they include the infant in the family tree. Examples of this are Beth and Alice who made a family portrait that includes the deceased child. They have portrayed the deceased child as a shadow. The photos show how the children belong to the family, but at the same time, they are different. In these ways, the transitional objects also function as ritualized acts, as described in our previous article (Værland et al., 2021). The bonds to the dead infant are tightened or loosened by the parents, and the objects help the families bring the infant out of liminality and into a status as a deceased family member.

Third, the transitional objects help the parents integrate the loss into their broader life history. According to trauma theory, if the trauma is powerful, the bereaved can find it difficult to integrate the event into their autobiographical histories (Blindheim, 2014). Losing an infant is a traumatic event, but unlike many other traumatic experiences, such as violence, abuse, injuries, and ill treatment, the experience is not exclusively negative. The child has given the parents a lot of joy and expectations during pregnancy. The parents also described the time they had together with their infant as a nice time, even if the child was stillborn or they knew they were about to lose it. This complexity of the trauma requires the bereaved to come to terms with the contrasting and non-complementary experiences of birth and death—often happening in close proximity. As previously mentioned, parents’ ongoing work with objects can be a way of integrating the trauma into their broader life story. The extent to which a traumatic experience leads to complicated grief reactions seems to be related to whether or not one is able to integrate the trauma into one’s autobiographical history (Nijenhuis, 2015). Physical items—such as those of the bereaved in this study—can help people interpret and process the trauma and assist them in gradually integrating the traumatic event into their broader life stories (Maddrell, 2013). When put on display, the items keep the memory of the lost child active for all members of the family. As such, transitional objects enable the bereaved to reframe the traumatic event and integrate it into their broader life history.

Fourth, transitional objects help the bereaved establish and maintain bonds with the deceased infant. The theory of continuing bonds states that the interaction between the deceased and the grieving remains (Klass, 2006). The physical absence of the deceased can be so strong that the absence is tangible. The deceased is not present physically but is still present through the emotions of the bereaved and physical objects. This became especially clear when the participants talked about the importance of the items the infant had close to the body. Clothes, blankets, and other things that had been on the baby’s body were of great significance for the bereaved as they brought the lost infant closer. The theory of continuing bonds describes grief as a relational and dynamic process of absence and presence (Maddrell, 2013). From the interviews, it is evident the parents moved different objects in and out of particular spaces. Objects can facilitate continuing bonds in different ways, as physical manifestations of the dead infant as well as tokens of the relation to the infant (Burgess et al., 2023). The bonds to the infant continue but also change as time passes. For instance, many participants mentioned that their approach to the transitional objects changed over time in various ways. An example of this is the teddy bear of the infant Belinda. It seems like the family that brings Belinda’s teddy bear with them are figuratively also bringing Belinda with them. However, there have been changes; the teddy bear is no longer brought everywhere. Although, when Belinda is in focus, when the family travels around and tells their story, the teddy bear is always there, almost living its own life. Another example is John, who explained how, in the beginning, he and his family spent 99% of their time looking at photos of their deceased child and talked about him. Now, they spend time at the grave but do not look in the memory box as much as they did in the beginning. Our interpretation of this dynamic relationship with memories has to do with a tightening and loosening of the bonds to the deceased child. The moving and making of a hierarchy of memories reinforces the transformation in the bereaved’s bonds with the dead child. The parents use the objects to symbolize an absent presence (Maddrell, 2013). Such ritualized acts, theoretically, can be understood as bereavement rituals (Mathijssen, 2018). The transitional objects, as symbols of the deceased infant, are transformed and incorporated in places that are close and more distant. For all, the bonds to the infant are more or less transformed. By moving the objects and preferences of some to the indifference of others, the parents transform their bonds with the infant (Mathijssen, 2018).

### Ambivalence Toward Transitional Objects

At the same times as the participants treasure transitional objects, they express ambivalence toward them. As we have seen, losing a child is a powerful traumatic experience (Christiansen et al., 2013), so ambivalence toward the objects is understandable. On the one hand, the objects remind the bereaved of their precious child. On the other hand, the physical objects remind the families of their shocking and traumatic loss of the child. According to all the participants, transitional objects are placed in different rooms. Some are displayed, others are more or less hidden, while others are stored in boxes almost all the time. The participants reported that the significance of the objects changed over time. Some of the objects are hard to look at just after an infant’s death, cf. Alice who said it took some time before she felt ready to look at photos of the baby on her phone. Other items were kept, even though they were not regarded as essential as time passed. Participants added that some objects were unproblematic to relate to, while others were more complicated, and time and emotions affected the experience.

Our study found that the ambivalence toward transitional objects arises because they often stir up good and painful emotions among the bereaved. The participants did not constantly want to confront these objects. They added that the objects had the potential to open and close the mourning process. Such objects facilitated the pain and helped the participants cry. Their habit of putting some of the objects out of sight was a way of taking agency over the mourning process and controlling the sorrow. This is in line with Stevenson et al. (2017), who show that parents can sometime choose when they want to confront themselves with their sorrow. One mother told us that when her children were not at home, she could choose to have a breakdown, as she preferred to confront herself with the sorrow when she was alone. Our participants also expressed that some objects were hard to face, as they provoked sorrow more than other objects. When participants allowed themselves to be confronted with these items and reacted, they were done with it for a while. When they closed a scrapbook, it was a way of putting their grief away and continuing life. In this way, the bereaved transform and negotiate their relation to the deceased (Mathijssen, 2018) and manage to gradually integrate the difficult memories.

### The Role of Healthcare Professionals in Memory Making: Executing Relational Autonomy

When analyzing our data, we became aware of the intervention from healthcare professionals to make memories on behalf of the parents. This theme was central in our previous article on memory making for bereaved parents (Værland et al., 2021). Retrospectively, the parents acknowledged the significance of these actions and were grateful the professionals had made memories on their behalf when they were not able to do so. This led us to reflect on relational autonomy theory.

When we first became aware of relational autonomy theory, we perceived the combination of the words relational and autonomy as a contradiction. How does the term relational go together with the widespread understanding of an autonomous human being as self-reliant and able to make choices based on own rationality? Our skepticism was informed by our Western understanding of autonomy, in which the self has been regarded traditionally as “[…] an autonomous, self-contained centre of thought and agency” (Davy, 2019, p. 101). This understanding of the self seems to underpin the understanding of autonomy in the four main principles of biomedical ethics which have influenced the field of medical ethics: beneficence, nonmaleficence, autonomy, and justice (Beauchamp & Childress, 2019). There is a common core understanding of the term autonomy as “self-government.” However, what this self-government entails remains controversial (Page, 2012; Varelius, 2006). The term autonomy is derived from the Greek words *autos* (self) and *nomos* (rule), and although the biomedical definition of autonomy has developed over the centuries, it has its roots in the Enlightenment and, in earlier times, was related to the German philosopher Immanuel Kant’s understanding of human beings as able to make choices based on their own rationality (Guyer, 2003). With these definitions of autonomy in mind, we saw a danger in reducing human beings to free individuals independent of others and to rational beings always capable of making choices based on own rationality. Although patient autonomy in the individualistic sense of the word stands strong in our time, there might also be a risk in this approach:

[…] there is a risk that by grounding nursing so firmly in a set of values based on the individual’s right to self-management, independence and autonomy, the nurse may develop the unfortunate preconception that patients always will, and can be active, self-managing and capable of making the right decisions for themselves on the basis of a number of choices. (Delmar, 2012, p. 241)

We became aware that there is no contradiction between dependence and independence and autonomy; only an individualistic conception posits a contradiction (Delmar, 2012). Relational autonomy states that “[…] autonomous beings are, of necessity, socially situated and interdependent” (Oshana, 2020, p. 1). According to Braut (2000), a human being’s autonomy and vulnerability varies throughout life, whereas the integrity of the human being is constant. This means human beings are autonomous and vulnerable at the same time. The shifting degree of autonomy and vulnerability throughout life does not meddle with a human being’s integrity.

Relational autonomy theory is often argued for in opposition to an individualistic understanding of autonomy which denies dependency as a key feature of human life (Mackenzie & Stoljar, 2000, pp. 3–31) and not as a theory standing on its own feet (Davy, 2019). However, “[…] relational autonomy is a rich and complex concept, formulated in complementary ways from diverse philosophical sources” (Gómez-Vírseda et al., 2019, p. 1). Our understanding of relational autonomy is derived from feminism, disability studies, the science of nursing, theology, and philosophy. Feminist theorists have developed an alternative definition of autonomy to a biomedical one. The social context and relations are seen as vital in developing the capacity for autonomy: “People are unable to develop into self-determining individuals without a nourishing environment or to be autonomous without support from others” (Davy, 2019, p. 104). Alvsvåg and Martinsen (2018) argue that we have been given life on the condition that we are vulnerable beings. They refer to the philosopher Knud E. Løgstrup and his book *Den etiske fordring* (The ethical demand), in which he radically claims that “a person can never be involved with another person without holding something of this other person’s life in their hands” (Løgstrup, 1956/1991, p. 25, Sviland’s translation). Løgstrup admits it is not easy to know what is at stake for another person or to know how to interpret and respond to a concrete situation. Nevertheless, the ethical claim calls on people to decide how they choose to act, and useful questions might be: “What does this claim actually mean? What does the other person need? What is actually going to be helpful for the other? What is our role, responsibility, and possibility in this situation?” (Sviland et al., 2022, p. 5). To balance between respect for peoples’ integrity, responsibility for one’s own self and care for others seems to be the core of relational autonomy: “The enablement of relational autonomy operates on at least two levels: helping the individual to negotiate the world around them and intervening in the social world to make it more accommodating of the individual” (Davy, 2019, p. 109).

We find relational autonomy theory adequate when trying to understand what is at stake between healthcare professionals and parents when objects are made on the initiative and insistence of the professionals. Books and other objects are made without systematic verifications of how the parents experience the process (Værland et al., 2021). However, the present contribution informs us how parents receive such items. We found that the objects were very important to all the parents we interviewed. Those who were persuaded by healthcare professionals to take photos of the child were grateful afterward, while those who were not encouraged to do so missed the opportunity of someone shouldering responsibility by taking photos. For many years, hospitals in Norway have had routines intending to help parents during the grieving period after a stillbirth (Christoffersen & Teigen, 2021). According to these authors, if the parents did not want to follow the professionals’ recommendations, the midwives would try to persuade them to make memories. This study and our research show that healthcare professionals take on a persuasive role in some cases. The professionals refer to research and former experience to convince parents to make what they believe is the right decision (Christoffersen & Teigen, 2021). We also find a kind of persuasiveness in the fact that the staff start to make memory books without asking the parents: “In a way, it is something that we inflict on them [the parents]” (Værland et al., 2021, p. 6). It is as if the professionals have the questions from Løgstrup in mind when they act on behalf of the parents (Sviland et al., 2022).

By intervening, the professionals help the parents preserve their autonomy in a situation of great vulnerability. They make transitional objects on behalf of the parents without waiting for the parents’ request to do so. In our understanding, this vicarious act is executed as an act of care, as: “[…] care for and by others is understood as constitutive of the very possibility of autonomous action in the social world” (Davy, 2019, p. 102). As the parents are in a state of shock and grief and not able to foresee what might help them later in their mourning process, the professionals use their knowledge and experience in their care for the parents. Underscoring such an intervention is the view that human beings are both dependent and autonomous at the same time (Alvsvåg & Martinsen, 2018; Løgstrup, 1956/1991).

Relational autonomy theory is thus adequate for gaining a deeper understanding of what is at stake when healthcare professionals make memories on behalf of parents who are in a situation that prevents them from thinking rationally about what will benefit them in their future bereavement work. This is in line with what the Australian disability researcher Laura Davy says about the role relations play in agency and autonomy: “[…] the agency and autonomy of individual persons can only emerge relationally, through the support and enablement of others” (Davy, 2019, p. 102). Both feminist and disability theorists warn against the risk of doing harm and emphasize the need to balance recognition of relationality, on the one hand, and respect for individual personhood, on the other hand. Underlying this negotiation is the contention that “[…] autonomy cannot be enabled without care, and care cannot be enabling without respect for autonomy” (Davy, 2019, p. 102). In our interpretation, the healthcare professionals’ insistence upon making memories can be understood as an act of care.

## Conclusion

In the following, we sum up our interpretation of the significance the objects have for the bereaved in our Norwegian context.

First, as transitional objects, they have a fourfold function for the bereaved: (i) Transitional objects comfort the bereaved when they absorb the fact that their child has gone from life to death. (ii) The objects are important elements in the bereaved’s ritualization around giving the child status as a family member, and at the same time acknowledging its status as deceased. (iii) The objects help the parents integrate the traumatic experience of loss into their broader life history. (iv) Transitional objects help the bereaved establish and maintain continuing bonds with the deceased infant.

Second, our data show that parents are ambivalent toward these objects. On the one hand, items are displayed, cherished, and regarded as a help for the bereaved. On the other hand, objects are kept away and avoided at times—as they stir up difficult emotions. Through the preference for some objects and indifference toward others, the parents transform their bonds to the lost infant.

Third, healthcare professionals’ intervention to make transitional objects on behalf of the parents is an act of care based on the professional’s knowledge and experience. The theoretical concept of *relational autonomy* is used here to understand what this intervention by healthcare professionals entails. When parents lose an infant, they are in a state of mind in which their vulnerability and dependence are more prominent than their autonomy. In such a situation, shock and grief prevent parents from making choices based on their own judgment and rationality. By making memories on behalf of the parents, healthcare professionals—who are in a professional relationship with the parents—preserve the parents’ integrity by elevating their autonomy. This is in our interpretation of an act of care, which can be understood in light of relational autonomy theory.

## Limitations

Some of the participants lost their infants during the COVID-19 pandemic and experienced restrictions on hospital visitations and the number of attendees at funerals. All the participants were Norwegian of origin, and the data is less heterogenous than what we had hoped for. While the participants in this study were “positive” toward the objects, other parents who do not attend bereavement or support groups may have different experiences. As this study is a qualitative one, the results cannot be generalized.
